# Chronic Cerebro-Spinal Meningitis

**Published:** 1873-06-15

**Authors:** 


### Original Communications.

### CHRONIC CEREBRO-SPINAL MENINGITIS.

I desire to call your attention to a class of cases of cerebro-spinal disease, concerning which I have seen little or nothing in our medical literature. The cases alluded to may be regarded as sequelæ of cerebro-spinal meningitis. It has been observed, both in my own practice and that of others with whom I have consulted, that many of the cases of the disease just named exhibited a strong tendency to be protracted in a chronic form ; or, more properly, when the acute symptoms had all disappeared, and convalescence was apparently complete, the patients did not progress in the recovery of their former flesh and strength ; on the contrary, they remained pale, cool, progressively emaciating, frequently tortured with neuralgic pains, and incapable of steadiness of muscular action or of locomotion in the upright position.

After the first three or four weeks the appetite often became good, the urinary secretion apparently natural, and the bowels nearly regular ; but the atrophy or emaciation continued; the hearing often remained dull; the pupils large; with morbid sensations in various parts, varying from mere creeping or formication to acute pains and momentary cramps. The latter are much the most frequent in the calves of the legs, the epigastrium, the head, neck and arms. But the most remarkable symptoms are those connected with attempts at co-ordination or progressive muscular motion in the upright position. There is no paralysis, for while in a horizontal position every voluntary muscle will respond to the mandate of the will. But the moment the patient assumes the upright position there is an unnatural feeling of giddiness or confusion in the head, with an imperfect control of the voluntary muscular system, varying in degree, in different cases, from a mere unsteady or staggering gait to such a complete tremor as to prevent the upright position altogether. I have now under my care, in the Mercy Hospital, a young woman who was attacked severely with cerebro-spinal meningitis three weeks since. At the end of two weeks all the active symptoms of disease had disappeared. The fever, the severe cephalalgia, the rigidity of the cervical muscles, etc., were entirely relieved, and the patient appeared convalescent. But instead of continuing her progress towards complete recovery, she remains now, at the end of the third week, pale in countenance, thin in flesh, cool in temperature, hearing impaired, pupils much larger than natural, pulse soft and weak, but nearly natural in frequency, mind clear, frequent shifting pains in the neck and head, and such a degree of impairment of the motor power in the upright position as to make her attempts to walk appear like one pretty thoroughly intoxicated. The following case will illustrate the most severe grade of symptoms seen in the class of cases under consideration:

Mr. ——, an intelligent laboring man, had a severe attack of cerebro-spinal meningitis, and was attended by one of our best and most experienced practitioners. He passed through the active stages of the disease, and put on the aspect of convalescence. He recovered so far as to be able to rise from his bed and make some attempts at walking—much like the case of the young woman just described—but subsequently retrograded so far as to be entirely unable to maintain the upright position.

It was eight or nine weeks after the primary attack that I was requested to see him in consultation with his attending physician ; and he was soon after removed to the hospital, where he came directly under my care. On examining the case with his attending physician, he presented the following symptoms: General aspect, anæmic, with considerable emaciation ; surface cool throughout ; pulse soft, weak, about natural in frequency; cardiac impulse rather feeble; sounds natural; respiration nearly natural; pupils much dilated; hearing slightly impaired; mind clear; appetite good ; bowels inactive; urinary secretions nearly normal in quantity and quality; but the slightest disturbance caused the eye-balls to tremble; and every attempt to raise him into an erect position produced such a violent and universal trembling, and agitation of the voluntary muscular system, that a return to the recumbent position, immediately, seemed necessary to avoid the supervention of general convulsions. He was subject to frequent shifting, neuralgic pains, and insomnia or indisposition to sleep at night. The patient had been in the condition here described already three or four weeks, undergoing no apparent change, except a gradual increase of the muscular trembling and helplessness. The dilated pupils, trembling of the eye-balls, and inability to maintain the erect position, had suggested to his physician the probability of effusion into the lateral ventricles of the brain, with a tendency to softening of cerebral structure. But from my observation of other cases, and from the fact that a degree of effusion sufficient to cause dilitation of the pupils and inability to maintain the erect position would almost necessarily induce coincident stupor and paralysis, instead of wakefulness and muscular tremor—except in infants, whose fontanelles and cranial sutures are still unclosed — led me to a different conclusion concerning the pathological condition of the cerebro-spinal portion of the nervous system.

Having met with several of these cases, presenting all degrees of severity in the symptoms, and studying carefully both the symptoms and the effects of remedies, I had been led to the conclusion that, on the subsidence of the primary disease, those parts of the brain and spinal cord involved had undergone such a molecular change, or rather such a change in the properties of the nerve substance, as to prevent the re-establishment of normal nutrition in the part.

Hence, instead of effusion, with or without a continuance of meningial inflammation in a chronic form, I regard the essential local condition of the cerebro-spinal structures involved as one of anæmia and impaired nutrition, tending to atrophy of the nerve matter. With this view, I advised the attending physician to keep the patient at rest in the recumbent position, give him a plain, easily-digestible diet, induce as much cheerful, mental quietude as possible, and give at each meal-time a table-spoonful of the following mixture :

℞.—Ext. malt (syrup), ℥ viii.

Comp. syrup hypophosphatis, ℥ iv.

Mix; and a single dose of Dover's powder and camphor, sufficient to produce sleep, at night. The advice was followed, and, as before stated, the patient was soon after removed to the hospital on account of insufficient accommodations in his boarding-house. In the hospital the treatment just stated was continued, with no important change, for three or four weeks, when the anodyne at night was omitted ; and at the end of six weeks the patient had recovered his flesh and strength sufficient to walk steady and firm, and left the hospital, claiming to be quite well. During the first week after he came into the hospital his improvement was so slight as to be hardly noticeable; but subsequently his progress was much more rapid.

The first few of these chronic cases that came under my observation were treated with counter-irritation; the bromides and iodides internally, aided by chloral, to relieve neuralgic pains, but with no improvement. I saw two cases that had been treated with a liberal use of alcoholic stimulants, opium and quinine; and had steadily grown worse instead of better. But since adopting the practice of treating this class of cases with rest, mild, nutritious food, and such remedial agents as are calculated to promote the nutrition of nerve structures, aided by just sufficient anodynes to procure rest at night, the results have been much more favorable. During the past two years I have met with three or four chronic cases presenting symtoms closely analagous to those above described, but which had not been preceded by any well marked attack of cerebro-spinal meningitis. On the contrary, they had been exposed to ordinary malarious influences for a considerable time. They all recovered slowly under the treatment above advised, with the addition of a moderate use of quinine.

---

A doctor in Iowa lately punctured a small pimple on a patient's nose with his vaccination lancet. It took beautifully.

